# Comparison of Two-Component Silyl-Terminated Polyether/Epoxy Resin Model and Complete Systems and Evaluation of Their Mechanical, Rheological and Adhesive Properties

**DOI:** 10.3390/polym14122421

**Published:** 2022-06-15

**Authors:** Ritvars Berzins, Remo Merijs-Meri, Janis Zicans

**Affiliations:** Institute of Polymer Materials, Faculty of Materials Science and Applied Chemistry, Riga Technical University, 3 Paula Valdena Street, LV-1048 Riga, Latvia; remo.merijs-meri@rtu.lv (R.M.-M.); janis.zicans@rtu.lv (J.Z.)

**Keywords:** silyl-terminated polyether, epoxy resin, two-component system, compatibilizer, mechanical, rheological and adhesive properties

## Abstract

The current research is devoted to the investigation of the influence of a secondary amine compatibilizer and customized additive package on the tensile, rheological and adhesive properties of a Silyl-terminated polyether (SIL)/Epoxy resin (EP) model and completed two-component systems. A SIL/EP model and completed two-component systems were developed over a broad range of the both pre-polymer ratios (90/10–30/70 wt.-to-wt%). Additive packages of the components A and B were designed to prevent premature polycondensation of the respective pre-polymers (including suitable catalysts for each of the pre-polymers, as well as vinyltrimetoxysilane as a drying agent for moisture control), to ensure easy processing and stable performance of the system. Results of the investigation testify that the values of the tensile strength and Shore-A hardness of the compatibilized systems are higher in comparison to unmodified ones. In the presence of the additive package, a further improvement of tensile strength and tensile strain values is observed for SIL-rich compositions (SIL content above 70 wt%), whereas at lower SIL concentrations, the reinforcing effect is considerably reduced. In respects to adhesion properties, the highest values to a broad range of substrates with different surface polarities are observed at the SIL/EP range from 80/20 to 50/50 wt.-to-wt%.

## 1. Introduction

Adhesives are considered as a special class of materials designed to adhere to the surfaces in contact ensuring their bonding at the development of a broad range of products for almost any sector of national economy. Among the materials with adhesive properties, polymer-based systems play an important role. Consequently, the polymer adhesives industry serves many of the important sectors in the world economy, including building and construction, transportation, electronics, packaging, sports and recreation, medicine and others. The value of the polymer adhesives market exceeded USD 43.7 billion in 2020 and is estimated to grow at a 5.3% compound annual growth rate between 2021 and 2025 [1]. Despite numerous commercial formulations developed, the offer of polymer-based adhesives with relatively high tensile strength values (tensile strength σ_break_ values over 3 MPa) is still limited. The performance of adhesives is largely determined by it formulation, including pre-polymers and additives used. In general, polymer adhesives are divided into one-component and two-component systems [2]. Most of the commercially available one-component adhesive systems, containing a certain active functional group, typically are superelastic (ultimate tensile elongation ε_break_ values > 100%) and are mostly used in the non-load bearing construction sector. To achieve higher tensile strength values, required in automotive, shipbuilding and structural engineering sectors, it is often necessary to use two-component systems. In respect to the pre-polymers used, acrylic, polyurethane, polyvinyl acetate, styrene, ethylene-vinyl acetate and epoxy adhesives are most common [1,2]. Polyurethane adhesives, available as both one-component and two-component formulations, are most widely used because of their ability to tailor properties of the final system over the broad range, simply by changing the ratio between the superelastic (polyol) and the rigid (isocyanate) elements of the macromolecule. Polyurethane adhesives are known for their short forming time necessary for the development of the strong bond, compatibility with a broad range of different materials and easy and clean handling, making them more advantageous over many other polymer adhesives. Polyurethane adhesives are used for a broad range of applications, including textiles, aerospace, food packaging, automotive, defense and other high-performance industry sectors, because of their excellent durable adhesion [3,4,5,6,7,8,9]. Currently, there is practically no alternatives for polyurethane systems as materials with high tensile strength values (σ_break_ > 3 MPa). However, the main disadvantages of polyurethane adhesives are environmental considerations due to the historically widespread use of diisocyanates for their manufacturing, the bubble-forming tendency of polyurethane adhesives during manufacturing, as well as the temperature sensitivity of the compositions due to strong H-bonds that makes their application difficult. To reduce occupational exposure to diisocyanates and, consequently, the cases of diisocyanate-induced asthma, which is recognized as a particular problem in C.A.S.E. (Coatings, Adhesives, Sealants, Elastomers) applications, a restriction on the use of diisocyanates was recently adopted under the REACH Regulation in the European Union, allocating a three-year transition period [10,11]. Consequently, in recent years, considerable attention has been attributed to the development of modified or hybrid two-component adhesives, based on the use of two or more pre-polymers to meet high-performance criteria by combining best properties of individual polymeric counterparts within a joint composite system. For example, by combining epoxy pre-polymer (EP) with a silyl-modified polyether (SIL), it is potentially possible to obtain materials with excellent toughness at a high strength and flexibility. In such a system, the role of the superelastic element is fulfilled by SIL [12,13,14], whereas the role of the rigid element is fulfilled by EP [15,16]. Unfortunately, in a recent review on the characterizing impact of the environment on the adhesion of sealed joints in facade applications performed by Nečasová and Liška, the presumption that hybrid silyl-modified polymer sealants are an appropriate replacement for polyurethane products was partly disproved due to a smaller stretchability of the selected silyl-modified products as well as a larger property change after artificial ageing. Consequently, there is a need to perform research on the development of new silyl-modified polymers and perspective hybrid systems based on silyl-modified polymers. However, there is shortage of detailed scientific information on SIL/EP systems, except for some research publications and patents [17,18,19]. In our previous publications, we have described some issues of the development of SIL/EP model systems (the systems which consisted only from pre-polymers and catalysts) without and with compatibilizers. Results of these investigations showed that N-(n-Butyl)-3-aminopropyltrimethoxysilane-compatibilized two-component SIL/EP systems demonstrated high tensile strength and strain values (over 6 MPa and 400%, respectively) [20,21], revealing the potential of the developed systems for creating novel commercial adhesive and sealant formulations with a broad range of mechanical properties. Unfortunately, for practical reasons and the economic viability of the finished product in the industry, it is usually necessary to tailor the cost–performance ratio of the product in respect to the target market. In the current research, attention is devoted to the investigation of the effects of the addition of a traditional standard additive package (comprising from plasticizers, fillers and other additives) to develop commercially viable completed systems, as well as to analyze their mechanical, rheological and adhesive properties. The inclusion of additives in the system not only makes it economically competitive, but also allows it to adjust its workability and proves that the finished product gains considerable practical advantages in operating in a two-component system, providing the required mechanical, adhesive and curing properties. It is expected that the developed SIL/EP systems are the first step to creating a competitive product for the adhesive and sealant market.

## 2. Materials and Methods

### 2.1. Materials

Materials, used for development of the investigated SIL/EP two-component model and completed systems, are summarized in Table 1, Table 2 and Table 3. The tables also reveal relative amounts of the materials necessary for development of the investigated two-component formulations at different SIL/EP ratios.

### 2.2. Preparation of A and B Components

A and B components were made by using 3-L laboratory mixer TEJA Engineering (Teja Engineering Sp. z o.o. Zabkowice Slaskie, Poland). First, all liquid raw materials (pre-polymers, plasticizers, compatibilizer/adhesion promoter, drying agent) were stirred for 5 min at 1000 rpm (central axis)/5 rpm (planetary axis), then fillers were added and the composition was stirred for 20 min at 3500 rpm (central axis)/35 rpm (planetary axis), then mixing was continued under vacuum for 30 min at 3500 (central axis)/35 (planetary axis) rpm.

Blends at the fixed SIL/EP ratios were mixed using SpeedMixer DAC 150 centrifugal laboratory mixer (FlackTek SpeedMixer, Landrum, SC, USA), casted in Teflon molds (dumbbell specimens) and cured at standard conditions—23 ± 2 °C, 50 ± 5% RH—for 1, 7 and 28 days.

### 2.3. Testing Methods

#### 2.3.1. Tensile Test

Tensile stress–strain measurements were done by using Zwick/Roell Z010 universal testing machine (ZwickRoell GmbH & Co. KG, Ulm, Baden-Württemberg, Germany). The tests were made according to ISO 527 at test speed 100 mm/min (dumbbell specimens). The testing was made for model and full systems after 1, 7 and 28 days of curing at 23 °C, 50% RH. The test result for each investigated system was reported as average from five parallel measurements. Standard deviation was evaluated by using Microsoft Excel software.

#### 2.3.2. Hardness Test

Hardness was tested according to ISO 7619 standard, using SCHMIDT PHPSA (Hans Schmidt & Co GmbH, Traunstein, Germany) equipment. The testing was made for model and full systems after 1, 7 and 28 days of curing at 23 °C, 50% RH. The test result for each investigated system was reported as average from five parallel measurements. Standard deviation was evaluated by using Microsoft Excel software.

#### 2.3.3. Oscillatory Shear Test

Viscosity, as well as elastic and viscous moduli measurements were performed by using Bohlin CVO 100 rheometer (Malvern Instruments, Inc. Westborough, MA, USA). The instrument was equipped with 20 mm diameter spindle ensuring plate–plate geometry (gap size 1000 µm). The tests at 25 °C were performed in oscillation mode—frequency 1 Hz and strain 0.006.

#### 2.3.4. Peel Test

Peel test was made using different substrates: alloy MS 63, stainless steel, copper, alloy 5005, epoxy fiberglass, EPDM (ethylene propylene diene monomer rubber) and PVC (polyvinyl chloride). The test was performed after 1, 7 and 28 days, with peeling of sealant from substrate (materials were stored at 23 °C, 50% RH). The test result for each investigated system was reported as average from three parallel measurements. Standard deviation was evaluated by using Microsoft Excel software.

#### 2.3.5. Lap Shear Test

Lap shear test was made using different substrates: stainless steel, polyvinylchloride (PVC) and wood (ash tree). The test was made according to EN 1465. Overlap area of the test specimens was 12.5 × 25.0 mm and the materials’ thickness was 0.2 mm. The test result for each investigated system was reported as average from five parallel measurements. Standard deviation was evaluated by using Microsoft Excel software.

## 3. Results and Discussions

### 3.1. Material Mechanical Properties

**Figure 1:**
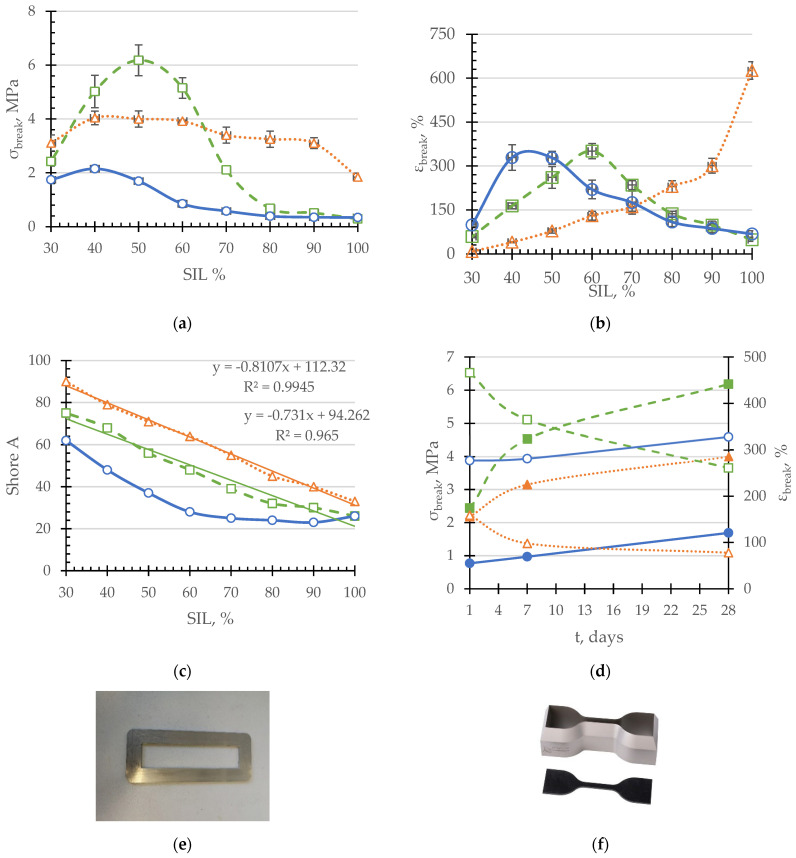
Tensile stress (**a**) and tensile strain (**b**) at break and Shore A hardness (**c**) of SIL/EP model systems (-○-), compatibilized model systems (-□-) and completed systems (-∆-) after 28 days of curing in standard conditions (T = 23 °C; RH = 50%), and the change of tensile stress (left axis, closed symbols) and tensile strain (right axis, open symbols) values during curing (**d**) of SIL50/EP50 compositions. The materials were formed into a metal frame (**e**) and cut out with a cutter (**f**) of a certain size, according to ISO 527 standard.

Mechanical properties of the investigated SIL/EP two-component systems are revealed in Figure 1. In comparison to the model systems, the tensile strength values of the complete systems are less dependent on the SIL/EP ratio, demonstrating a leveling off over the broad range of SIL/EP ratios (SIL/EP 30–90 wt.-to-wt%), which may be explained by the effect of the addition of the filler and plasticizer package. The effect of the rigid-chain EP addition on the 3D structure of the compatibilized SIL/EP system may be evaluated by comparing the tensile strength values of SIL/EP 90/10 composition with those of neat SIL. As it is demonstrated, the addition of 10 wt% of EP ensures a 50% increment in the tensile strength value of the complete system. Interestingly, those tensile strength values of the complete systems are further increased only to a limited extent (by 30%) by raising the concentration of EP in the system from 10 wt% to 60 wt%. In the case of unfilled systems, the addition of EP moieties only becomes effective above 20 wt% for compatibilized SIL/EP formulations and above 40 wt% for unmodified compositions. Evidently, this is because of the increased interaction between the base pre-polymers’ tensile strength of the compatibilized systems which considerably exceeds that of the unmodified compositions.

**Figure 2:**
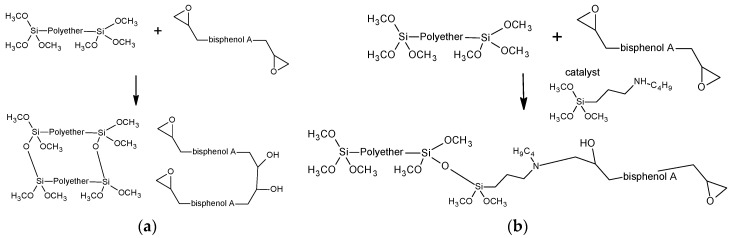
Reaction scheme of two-component SIL/EP system ((**a**) without compatibilizer, (**b**) with compatibilizer).

The possible mechanism of interaction between SIL and EP pre-polymers in the presence of a compatibilizer is demonstrated in Figure 2. It is worth noting that within the EP concentration range 40–60 wt.-to-wt%, the tensile strength values of the unfilled compatibilized systems are even greater than those of the complete systems. Evidently, in the presence of a filler/plasticizer package, the effect of the compatibilizer is somewhat reduced, leading to a decreased interaction between SIL-EP pre-polymers due to the fact that, because of the decreased macromolecular flexibility, access to possible interaction sites is reduced. Consequently, the maximum tensile strength values are reached at 50 wt% of EP for compatibilized, but unfilled systems (absolutely the highest values) and at 60 wt% of EP for filled and compatibilized systems and unfilled/uncompatibilized systems (for the latter, the lowest tensile strength value is observed). If the weight content of EP is decreased, the decrement of the tensile strength values begins due to the excessive concentration of rigid segments within the system (Figure 1). However, it is important to mention that fillers and plasticizers manipulate the workability of two-component systems, making it possible to create finished products with a broad range of rheological properties, starting from low-viscosity fluids and ending with thick pastes. Although they are important as reference points, adhesive and sealant compositions, which consist of only polymers, are usually without any significant practical use because of workability problems and high-costs.

Tensile strain at the break of SIL is considerably larger than that of an unfilled system due to the additive package introduced, especially because of the effect of a non-phthalate plasticizer. By increasing the content of rigid EP groups, strain at the break values ε_break_ consequently decreases. The most rapid change in strain at the break value is observed upon introduction of the first 10 wt% of EP, similarly as it was in the case of tensile stress at the break. The fact that the strain at the break values decreases and is almost constant with the stress at the break values within the EP concentration ranging from 10 to 60 wt% is explained by earlier-occurring strain hardening, initiated by the presence of the plasticizer/filler package on the one hand, and an increasing amount of rigid EP moieties on the other hand. By comparing ε (SIL) relationships of the completed and the model systems, either uncompatibilized or compatibilized, one can observe similar trends to σ (SIL) relationships. Up to a 20 wt% of EP content, the values of strain at the break of the model systems are considerably lower than those of the completed system, whereas no considerable differences are observed between uncompatibilized and compatibilized compositions. Above an EP content of 20 wt%, the values of compatibilized model systems become considerably larger than those of uncompatibilized compositions, due to increased interactions between the pre-polymers. Above an EP content of 40 wt%, increased interactions between the pre-polymers results in reduced flexibility. Without the compatibilizer, the maximum strain at the break values of the model systems is shifted to the direction of higher EP values (60 wt%).

Stress–strain characteristics describe materials’ properties in bulk, however, for the evaluation of the practical applicability of the developed adhesives and sealant systems, surface hardness plays an important role. The surface hardness of the developed two-component systems, expressed as Shore-A hardness, is depicted in Figure 1c. In contrast to the tensile properties, the Shore-A hardness of the investigated compositions is characterized by distinct, smoother relationships as a function of the EP/SIL content. This is most probably related to the different arrangement of macromolecules in the surface layers and bulk due to the fact that the hardening of the compositions starts from the external surface of the test specimens. As expected, the highest Shore-A hardness values are for formulations of the completed systems, greatly because of the reinforcing effect of the filler/plasticizer system used. In addition, the Shore-A hardness values of both compatibilized systems almost linearly increase by increasing the EP content, which is not true for the unmodified model systems. By considering that experimental data of the completed systems demonstrate the best fit with a linear trendline, it is possible to judge that the additive package used also contributes to the increased compatibility within the SIL/EP system. By evaluating hardening kinetics, one can observe that the process of the development of the 3D network in the compatibilized systems is completely different in comparison to SIL/EP compositions without a compatibilizer. According to the proposed cross-linking scheme, depicted in Figure 2, in the case of the unmodified SIL/EP compositions, the extension of SIL and EP pre-polymers occur without remarkable mutual interactions, leading to a gradual increment of both σ_break_ and ε_break_ within the investigated curing time growth interval. In the case of compatibilized systems, a more intense development of the cross-linked structure is observed due to increased interactions between both the pre-polymers, especially at smaller curing time values (up to 7 days). At higher curing times (7 to 28 days), the hardening process is reduced due to the decreased number of active unoccupied potential cross-linking sites. It is also observed that, in the presence of the additive package introduced, the tensile strength increment of the completed system occurs faster than in the case of the compatibilized model system, denoting to the effective reinforcement of the system.

### 3.2. Material Rheological Properties

**Figure 3:**
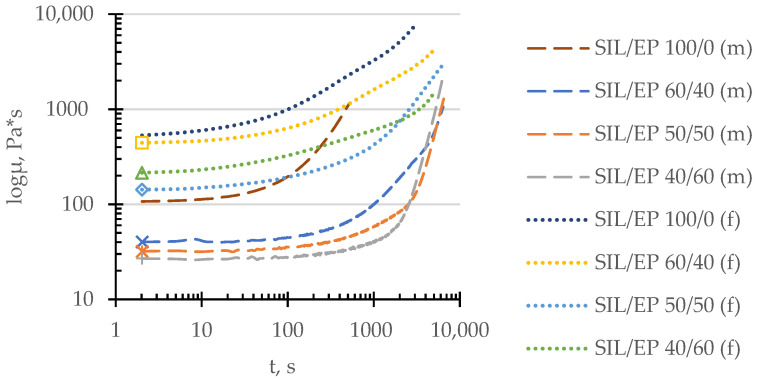
Viscosity growth in time of compatibilized model (m) and completed (f) systems, following SIL/EP wt.-to-wt. ratios: 100/0 (…/---), 60/40 (□/x), 50/50 (∆/✴) and 40/60 (◊/+).

The viscosity–time relationships of selected SIL/EP two-component systems are depicted in Figure 3, where the rheological behavior of the completed and model systems ensuring the highest mechanical properties is compared with that of the SIL pre-polymer. The analysis allows one to compare the curing behavior of the completed systems with that of model systems and the SIL pre-polymer. As already expected, the initial viscosity values of all the completed systems are higher, mostly due to the presence of rigid fillers within the system. By the introduction of EP moieties, the initial viscosity values of both the model and the completed systems start to decrease due to the lower viscosity of the used EP resin (11–14 Pa s, according to the material data sheet). It is worth mentioning that it is the case of the completed systems that the viscosity drop is less pronounced due to the presence of the additive package. The effect of the addition of the EP group is more clearly observed in the case of the model systems. Additionally, one can observe that upon introduction of the EP groups in the compositions, the curing speed of the model systems is considerably decreased, which could be regarded also as an advantage, while more time is available for a qualitative workout at the construction site. In the case of the completed systems, the hardening time is decreased to a certain amount of time in comparison to the model systems due to the presence of the additives, shifting the material hardening balance to the direction of lower time values.

**Figure 4:**
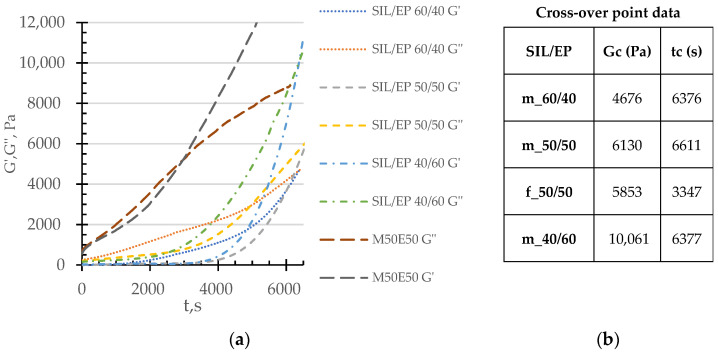
Elastic and viscous moduli growth in time (**a**) and corresponding cross-over point data (**b**) of completed (f) and compatibilized model (m) systems, following SIL/EP wt.-to-wt. ratios: 60/40 (dot dashed line), 50/50 (dashed line) and 40/60 (dotted line).

In Figure 4 the changes of elastic and viscous moduli in the time of the selected model and completed systems of SIL/EP are illustrated. As it was demonstrated in our previous publication [21], the modification of the investigated SIL/EP model systems by a secondary amine group containing a compatibilizer was effective in the increasing of tensile strength at considerable ultimate deformations, revealing that the gelling time was already at 6400 s. In the case of the investigated completed systems, the form of viscoelastic relationships becomes more complicated due to individual contributions of each of the additives. As it is evident from Figure 4, upon the introduction of the additive package, the system becomes more structured, mostly due to the development of the skeleton network of CaCO_3_ particles, resulting in the increased values of both storage and viscous moduli. Consequently, as demonstrated in the case of the completed SIL/EP 50/50 system, the determination of the G′–G″ cross-over point becomes more difficult. However, the trend is that the cross-over point is shifted towards the direction of lower time values.

### 3.3. Material Adhesion Properties

#### 3.3.1. Material Peel Test

The adhesive properties of the completed systems are characterized by the peel test and the lap shear test using several industrially important substrates which are summarized in Table 4. The peel test was performed in the whole range of the investigated SIL/EP ratios from 100/0 to 30/70. Based on the results of the peel test, three SIL/EP pre-polymer ratios (100/0, 80/20 and 50/50) were chosen for the lap shear test using three substrate types with different surface energies, namely, wood (56 mJ/m^2^), stainless steel (45 mJ/m^2^) and PVC (35 mJ/m^2^), in a decreasing order of it polarities.

The results of the peel test allow one to conclude that at certain SIL/EP ratios, adhesion to all of the tested substrates is improved. Even after 1 day of curing, certain systems within the SIL/EP ratio range of 90/10 to 60/40 demonstrate good adhesion to most of the substrates. By increasing the curing time, adhesion to most of the substrates is increased and, consequently, after 28 days of curing, a 100% cohesive failure is observed for almost all the investigated systems within the mentioned SIL/EP ratio range. This adhesion improvement can be explained by the fact that a compatibilized multi-component system, consisting of constituents with various polarities, has a greater number of potentially active sites capable for interaction with substrates of different surface energies. The decrement of adhesion of the completed systems with more than 50–60 wt% of EP pre-polymer occurs because of the brittlening of the material, as demonstrated also by tensile tests.

#### 3.3.2. Material Lap Shear Test

Results of the lap shear tests (Table 5), in general, confirm the results of the peel test, demonstrating that the neat SIL, as well as the selected formulations of the completed systems, show good adhesion to stainless steel and wood, whereas the adhesion to PVC is worse. It is worth mentioning that adhesion to all the mentioned substrates increases by increasing the content of the EP pre-polymer in the system; in addition, the adhesive strength is increased but there is ultimate deformation—decreased denoting to increased brittleness.

## 4. Conclusions

Results on the effects of the compatibilizer addition on the properties of SIL/EP two-component systems lead to conclusions that, in comparison to the uncompatibilized counterparts:

- The tensile strength and Shore-A hardness of the secondary amine compatibilizer containing systems are larger due to the increased interactions between the pre-polymers; the tensile strength of the compatibilized system may be increased up to 187% after 28 days of curing.
- The development of the 3D structure in the compatibilized systems occurs faster and is more efficient because the compatibilizer promotes the formation of a joint SIL/EP network with a higher molecular mass, as well being a result of the addition of the additive package.
- Shore-A hardness values of the developed completed two-component systems are further increased within the whole range of the investigated SIL/EP ratios, whereas the tensile strength and strain at the break values are increased only for the SIL matrix compositions, because fillers structurize elastic SIL polymers.
- Due to structural confinements, the tensile strength and strain at the break values of the completed systems at a SIL content below 70 wt% decrease below the level achieved by the compatibilized model systems.
- The maximal values of tensile strength of the completed two-component system are reduced in comparison to the model system, yet the tensile strength values are smoothed over a wide range, which increases the robustness of the material.
- It is possible to tailor the rheological properties of the investigated systems with plasticizers and fillers to ensure the necessary workability to a specific application, as well as to raise the consumer friendliness of finished adhesives and sealants.
- The viscosities of the completed systems are increased, but the hardening times are decreased because the amount of inert raw materials in the system increasing and the number of reactive elements decreasing.
- Adhesion to a broad range of substrates with different surface polarities (PVC, alloy 5005, stainless steel, copper, alloy MS 63) is increased with the added epoxy resin content, demonstrating advantages of hybrid composition due to the presence of EP resin bisphenol A moieties which, in combination with SIL fragments, are able to form adhesion to additional substrates.

In general, two-component systems, developed in the framework of this research, demonstrate a broad range of mechanical and adhesion properties, revealing a potential to be suitable cost-efficient alternatives (together with special additives) for construction, automotive, shipbuilding and other markets.

Due to the decreased tensile properties, particularly ultimate deformation, of the completed systems below a SIL content of 70 wt%, research on the optimization of the most appropriate formulation of filler package should be continued by changing the concentration of existing rigid fillers or integrating other functional fillers in the system.

## Figures and Tables

**Table 1:** Characterization of two-component SIL/EP formulations (model system without compatibilizer).

Trade Name	Manufact.	Chemical Structure	Function	g Per the Denoted wt.-to-wt. Ratio of SIL/EP							
				100/0	90/10	80/20	70/30	60/40	50/50	40/60	30/70
A component											
SAX 520	Kaneka Belgium NV, Westerlo-Oevel, Belgium	Silyl-terminated polymer	Pre-polymer	100	90	80	70	60	50	40	30
Lupragen N600	BASF, Ludwigshafen, Germany	N,N′,N″-tris-(dimethylaminopropyl)hexahydrotriazine	Catalyst	0	0.2	0.4	0.6	0.8	1	1.2	1.4
B component											
D.E.R. 331	The Dow Chemical Company, Midland, MI, USA	Epoxy resin	Pre-polymer	0	10	20	30	40	50	60	70
Tibcat 216	TIB Chemicals AG, Mannheim, Germany	Dioctyltin dilaurate (DOTL)	Catalyst	0.2	0.18	0.16	0.14	0.12	0.1	0.08	0.06
Water	-	Water	Catalyst	0.67	0.6	0.53	0.46	0.39	0.32	0.25	0.18

**Table 2:** Characterization of two-component SIL/EP formulations (model system with compatibilizer).

Trade Name	Manufact.	Chemical Structure	Function	g Per the Denoted wt.-to-wt. Ratio of SIL/EP							
				100/0	90/10	80/20	70/30	60/40	50/50	40/60	30/70
A component											
SAX 520	Kaneka Belgium NV, Westerlo-Oevel, Belgium	Silyl-terminated polymer	Polymer	100	90	80	70	60	50	40	30
Dynsylan 1189	Evonik Industries AG, Essen, Germany	N-(n-Butyl)-3-amino propyltrimethoxysilane	Compatibi-lizer/adhesion promoter	1.5	1.5	1.5	1.5	1.5	1.5	1.5	1.5
Lupragen N600	BASF, Ludwigshafen, Germany	N,N′,N″-tris-(dimethylaminopropyl)hexahydrotriazine	Catalyst	0	0.2	0.4	0.6	0.8	1	1.2	1.4
B component											
D.E.R. 331	The Dow Chemical Company, Midland, MI, USA	Epoxy resin	Polymer	0	10	20	30	40	50	60	70
Tibcat 216	TIB Chemicals AG, Mannheim, Germany	Dioctyltin dilaurate (DOTL)	Catalyst	0.2	0.18	0.16	0.14	0.12	0.1	0.08	0.06
Water	-	Water	Catalyst	0.67	0.6	0.53	0.46	0.39	0.32	0.25	0.18

**Table 3:** Characterization of two-component SIL/EP formulations (completed system).

Trade Name	Manufact.	Chemical Structure	Function	g Per the Denoted wt.-to-wt. Ratio of SIL/EP							
				100/0	90/10	80/20	70/30	60/40	50/50	40/60	30/70
A component											
SAX 520	Kaneka Belgium NV, Westerlo-Oevel, Belgium	Silyl-terminated polymer	Polymer	40	36	32	28	24	20	16	12
Hexamoll DINCH	BASF, Ludwigshafen, Germany	1,2-Cyclohexane dicarboxylic acid	Non-phtalate plasticizer	15	13.50	12	10.50	9	7.50	6	4.50
Dynsylan 1189	Evonik Industries AG, Essen, Germany	N-(n-Butyl)-3-amino propyltrimethoxysilane	Compatibi-lizer/adhesion promoter	1	1	1	1	1	1	1	1
Omycarb 1T	Omya AG, Oftringen, Switzerland	Ground CaCO_3_	Filler	17.13	15	13.8	12	10.30	8.60	6.80	5.00
Hakuenka CCR-S10	Omya AG, Oftringen, Switzerland	Precipitated CaCO_3_ coated with fatty acids	Filler	25	22.5	20	17.5	15	12.5	10	7.5
Dynasilan VTMO	Evonik Industries AG, Essen, Germany	Vinyltrimethoxysilane	Drying agent	1	0.9	0.8	0.7	0.6	0.5	0.4	0.3
Lupragen N600	BASF, Ludwigshafen, Germany	N,N′,N″-tris-(dimethylaminopropyl)hexahydrotriazine	Catalyst	0	0.62	1.33	2	2.66	3.33	4	4.6
B component											
D.E.R. 331	The Dow Chemical Company	Epoxy resin	Polymer	0	4	8	12	16	20	24	28
Hexamoll DINCH	BASF, Ludwigshafen, Germany	1,2-Cyclohexane dicarboxylic acid	Non-phtalate plasticizer	0	1.5	3	4.5	6	7.5	9	10.5
Omycarb 1T	Omya AG, Oftringen, Switzerland	Ground CaCO_3_	Filler	0	1.7	2.38	3.69	4.92	6.14	7.45	8.76
Hakuenka CCR-S10	Omya AG, Oftringen, Switzerland	Precipitated CaCO_3_ coated with fatty acids	Filler	0	2.5	5	7.5	10	12.5	15	17.5
Tibcat 216	TIB Chemicals AG, Mannheim, Germany	Dioctyltin dilaurate (DOTL)	Catalyst	0.2	0.18	0.16	0.14	0.12	0.1	0.08	0.06
Water	-	Water	Catalyst	0.67	0.6	0.53	0.46	0.39	0.32	0.25	0.18

**Table 4:** Peel tests of two component SIL/EP completed system (C—cohesive, A—adhesive fracture failure).

Substrate	Days	SIL/EP (%)							
		100/0	90/10	80/20	70/30	60/40	50/50	40/60	30/70
Alloy MS 63	1	**A100**	**C70A30**	**C100**	**C100**	**C100**	**C100**	**C50A50**	**A100**
	7	**C70A30**	**C100**	**C100**	**C100**	**C100**	**C100**	**C100**	**A100**
	28	**C100**	**C100**	**C100**	**C100**	**C100**	**C100**	**C100**	**A100**
Stainless steel	1	**A100**	**A100**	**A100**	**C100**	**C100**	**C100**	**C10A90**	**A100**
	7	**A100**	**C100**	**C100**	**C100**	**C100**	**C100**	**C20A80**	**A100**
	28	**C100**	**C100**	**C100**	**C100**	**C100**	**C100**	**C50A50**	**A100**
Copper	1	**A100**	**C100**	**C100**	**C100**	**C100**	**C100**	**C20A80**	**A100**
	7	**C80A20**	**C100**	**C100**	**C100**	**C100**	**C100**	**C30A70**	**A100**
	28	**C100**	**C100**	**C100**	**C100**	**C100**	**C100**	**C50A50**	**A100**
Alloy 5005	1	**A100**	**C90A10**	**C100**	**C100**	**C100**	**C100**	**C100**	**A100**
	7	**A100**	**C100**	**C100**	**C100**	**C100**	**C100**	**C100**	**A100**
	28	**C100**	**C100**	**C100**	**C100**	**C100**	**C100**	**C100**	**A100**
Epoxy fiberglass	1	**C100**	**C100**	**C100**	**C100**	**C100**	**C100**	**C100**	**A100**
	7	**C100**	**C100**	**C100**	**C100**	**C100**	**C100**	**C100**	**A100**
	28	**C100**	**C100**	**C100**	**C100**	**C100**	**C100**	**C100**	**A100**
EPDM	1	**C100**	**C100**	**C100**	**C100**	**C100**	**A100**	**A100**	**A100**
	7	**C100**	**C100**	**C100**	**C100**	**C100**	**A100**	**A100**	**A100**
	28	**C100**	**C100**	**C100**	**C100**	**C100**	**A100**	**A100**	**A100**
PVC	1	**A100**	**A100**	**A100**	**C70A30**	**C100**	**C100**	**A100**	**A100**
	7	**A100**	**C80A20**	**C100**	**C100**	**C100**	**C100**	**A100**	**A100**
	28	**A100**	**C100**	**C100**	**C100**	**C100**	**C100**	**A100**	**A100**

**Table 5:** Lap shear tests of two component SIL/EP-completed systems.

Substrate	Days	Paramater	SIL/EP Ratio (wt.-to-wt%)		
			100/0	80/20	50/50
PVC	28	σ_break_ [MPa]	0.09	0.21	0.25
		ɛ_break_ [%]	13	9	8
		Type of the fracture	**A100**	**C20A80**	**C30A70**
Stainless steel	28	σ_break_ [MPa]	1.42	5.5	6.2
		ɛ_break_ [%]	124	14	10
		Type of the fracture	**C100**	**C100**	**C100**
Wood (ash)	28	σ_break_ [MPa]	1.6	4.3	4.7
		ɛ_break_ [%]	210	30	15
		Type of the fracture	**C100**	**C100**	**C100**

## Data Availability

The data presented in this study are available on request from the corresponding author.

## References

[1] Global Adhesives Market Size by Technology. https://www.gminsights.com/industry-analysis/adhesives-and-sealants-market-report.

[2] Comparing One-Component and Two-Component Adhesives. https://www.chemical-concepts.com/blog/2018/02/comparing-one-component-two-component-adhesives/.

[3] One-Component Polyurethane Adhesives and Sealants. https://www.bostik.com/uk/en/our-adhesive-technologies/one-component-polyurethane-adhesive/.

[4] Polyurethane Adhesives and Sealants. https://www.adhesiveplatform.com/polyurethane-adhesives-and-sealants/.

[5] Sahooa S., Mohanty S., Nayaka K. (2018). Biobased polyurethane adhesive over petroleum based adhesive: Use of renewable resource. J. Macromol. Sci. Part A Pure Appl. Chem..

[6] Bockel S., Harling S., Konnerth J., Niemz P., Weiland G., Hogger E., Pichelin F. (2020). Modifying elastic modulus of two-component polyurethane adhesive for structural hardwood bonding. J. Wood Sci..

[7] Quinil J.S., Marinucci G. (2012). Polyurethane structural adhesives applied in automotive composite joints. Mater. Res..

[8] Jiang W., Hosseinpourpia R., Biziks V., Ahmed S.A., Militz H., Adamopoulos S. (2021). Preparation of Polyurethane Adhesives from Crude and Purified Liquefied Wood Sawdust. Polymers.

[9] Garmann H., Puck C.H., Mosshammer G. (2006). High-Strength Polyurethane Adhesive. U.S. Patent.

[10] Commission Regulation (EU) 2020/1149 of 3 August 2020 Amending Annex XVII to Regulation (EC) No 1907/2006 of the European Parliament and of the Council Concerning the Registration, Evaluation, Authorisation and Restriction of Chemicals (REACH) as Regards Diisocyanates (Text with EEA Relevance). https://eur-lex.europa.eu/legal-content/EN/TXT/?qid=1596534449847&uri=CELEX:32020R1149.

[11] Rother D., Schlüter U. (2021). Occupational Exposure to Diisocyanates in the European Union. Ann. Work Exposures Health.

[12] Barfurth D., Mack H. (2004). Silane Formulation for Moisture-Crosslinking Hybrid Adhesives and Sealants.

[13] Gadhave R.V., Gadhave C.R., Dhawale P.V. (2021). Silane Terminated Prepolymers: An Alternative to Silicones and Polyurethanes. Open J. Polym. Chem..

[14] Yuan Y., Zhang Y., Fu X., Jiang L., Liu Z., Hua K., Wu B., Lei J., Zhou C. (2016). Silane-terminated polyurethane applied to a moisture-curable pressure-sensitive adhesive using triethoxysilane. RSC Adv..

[15] Cardoso M., Pinto J., Campilho R., Nóvoa P.J.R.O. (2020). A new structural two-component epoxy adhesive: Strength and fracture characterization. Procedia Manuf..

[16] Rudawsk A. (2021). Mechanical Properties of Selected Epoxy Adhesive and Adhesive Joints of Steel Sheets. Appl. Mech..

[17] Nečasová B., Liška P. (2020). Research Summary on Characterizing Impact of Environment on Adhesion of Sealed Joints in Facade Applications. Materials.

[18] Devroey D.R.E., Homma M. (2021). Blends of silyl-terminated polyethers and epoxides as elastic adhesives. Int. J. Adhes. Adhes..

[19] Zeng D., Liu Z., Zou L., Wu H. (2021). Corrosion Resistance of Epoxy Coatings Modified by Bis-Silane Prepolymer on Aluminum Alloy. Coatings.

[20] Bērziņš R., Merijs-Meri R., Zicāns J. (2018). Effect of Amine Containing Compatibilizers on Mechanical and Rheological Properties of a Two-Component Silyl-Terminated Polyether/Epoxy Resin System. Proc. Est. Acad. Sci..

[21] Bērziņš R., Merijs-Meri R., Zicāns J., Loca D. (2017). Compatibilizers Effect on Silyl-Terminated Polyether/Epoxy Resin System Mechanical and Rheological Properties. Key Engineering Materials, Proceedings of the Engineering Materials and Tribology XXV, Riga, Latvia, 3–4 November 2016.

